# A diachronic corpus-based study on the construction of regional image in Western mainstream media—a case analysis of Henan province in China

**DOI:** 10.3389/fpsyg.2025.1645226

**Published:** 2026-01-12

**Authors:** Manyu Zhang

**Affiliations:** School of Foreign Languages, Henan Medical University, Xinxiang, China

**Keywords:** diachronic corpus, media discourse, regional image, text mining, transitivity system

## Abstract

Drawing on a diachronic corpus of Henan-related reports from 2006 to 2025, this study examines how the province is discursively constructed in Western mainstream media by integrating text mining with a transitivity-based social role analysis. Leximancer 5.0 was employed to map semantic themes across the two periods, and material process clauses were analyzed to identify Henan’s Actor-Goal configurations within the transitivity framework. The thematic analysis reveals both continuity and change, with crisis-oriented themes continuing to dominate, accompanied by a shift toward urban-economic themes and reduced cultural visibility. The transitivity analysis reveals a pronounced diachronic shift in Henan’s social-role configuration, as it transitions from being primarily Goal-positioned in 2006–2015 to predominantly Actor-positioned in 2016–2025, with the dominant Actor role shifting from Destructor to Doer and the Helped role declining substantially, which together strengthen Henan’s discursively constructed agency. Overall, the findings illustrate how the combined analysis of thematic patterns and transitivity-based role configurations can trace the diachronic evolution of Henan’s discursive representation in Western media.

## 1 Introduction

Henan, situated in the core of China’s Central Plains, occupies a historically and strategically significant position within China’s territorial and cultural landscape. As the cradle of Chinese civilization and one of the country’s most populous provinces, Henan has long played a central role in political, cultural, and economic development. In the contemporary era, it functions as a major transportation hub and a key node in the Belt and Road Initiative. Given this combination of historical depth and strategic relevance, enhancing Henan’s international visibility has become increasingly important in the context of globalization. In this broader process of international engagement, understanding how Henan’s image is constructed is particularly crucial. Regional images consist of both self-constructed narratives shaped by domestic actors and other-constructed representations produced by external observers (Sun, 2002). As Morgenthau (1985) notes, how others perceive a region can be just as important as what it “really” is. Although other-constructed images may be selective or distorted, they often exert significant influence over a region’s global standing and identity.

To systematically examine how the other-constructed image of Henan has taken shape over time, this study focuses on the period from 2006 to 2025, a span that captures critical shifts in both Henan’s development policy and international positioning. The two-decade period aligns with the four successive Five-Year Plans (from the 11th to the 14th), which reflect staged transformations in the province’s economic and social priorities. Within this broader developmental trajectory, a significant turning point occurred in March 2015, when Henan was designated as an inland hub in China’s Belt and Road Initiative, substantially elevating its national strategic role. This shift was reinforced in March 2016, when the provincial government recognized the 12th Five-Year period as a milestone of scientific planning, innovation, and image enhancement. In light of these developments, the study divides the corpus into two comparative phases, with 2006–2015 period characterized by Henan’s traditional development, and 2016–2025 period characterized by its modern transformation.

Despite this developmental trajectory, Henan continues to face persistent challenges in shaping its international image, which has long been constrained by regional prejudice and an enduring image crisis abroad (Liu and Hai, 2022). To capture how Western mainstream media have represented Henan over time and how these representations have changed, the present study adopts a dual analytical framework integrating text mining with transitivity analysis. Text mining is employed to identify diachronic shifts in semantic themes, while the transitivity system enables a systematic examination of discursive patterns of “Henan does what to X” and “X does what to Henan,” thereby revealing the social roles and agency configurations assigned to the province. By applying this integrated approach to a 20-year corpus (2006–2025), the study links thematic developments with their grammatical realizations, thereby offering a deeper and more nuanced account of the evolving international image of Henan. In line with this research design, the study is guided by the following two research questions:

1. How does the thematic representation of Henan differ between 2006–2015 and 2016–2025?
2. How does the transitivity-based construction of Henan’s social roles differ between 2006–2015 and 2016–2025?

## 2 Literature review

The concept of the “city image” was first introduced by Lynch (1960), who emphasized that it is shaped through a bilateral process involving both the physical environment and human perception. While Lynch’s framework highlights the interaction between external spatial conditions and individual cognitive mapping, personal impressions alone do not constitute a stable or widely accepted regional image. Media discourse therefore plays a crucial role in mediating between physical reality and collective cognition, constructing more coherent and socially recognizable representations of regions (Jiang and Kuang, 2023). Through such representational practices, the media organize information, foreground particular attributes, and shape public perceptions of regions, making discourse analysis essential for examining how regional images are formed and circulated. Existing research on regional images in media discourse adopts diverse analytical approaches, which can broadly be conceptualized in terms of what media discourse represents about regions and how it discursively constructs regional meanings.

As for what media represent about regions, scholars typically employ content analysis or corpus-based quantitative methods to examine thematic distributions, reporting frequencies, sentiment orientations, and distributional patterns of coverage. Content analysis has been widely used to map the thematic and evaluative tendencies of regional reporting, as illustrated by studies of Brussels (Wiard and Pereira, 2018), Suzhou (Jiang and Kuang, 2023), and Henan in British media (Qin, 2019). Corpus-driven investigations, including studies on Jiangxi (Lian et al., 2024), Yiwu (Lu et al., 2022), and Shaoxing (Lou, 2022), examine regional portrayals by analyzing lexical frequency distributions, collocational patterns and other quantitative features of news discourse. While these studies illuminate the informational structure and overall tendencies of regional coverage, they primarily identify recurrent patterns of representation and offer limited insight into the linguistic mechanisms through which evaluative meanings and social representations are realized.

As for how regional images are discursively constructed in media discourse, prior studies typically draw on corpus-assisted critical discourse analysis to examine discourse bias and ideological positioning, as seen in research on Hong Kong (Zhou, 2025), Harbin (Yan, 2024), and Henan (Wei and Wu, 2024). While informative, this line of work remains largely interpretive and offers limited insight into the linguistic realization of experiential meanings. To address this limitation, some scholars have turned to Systemic Functional Linguistics, particularly the transitivity system, which provides a systematic framework for analyzing how actions, participants, and experiential meanings are encoded in news discourse. Studies on Qingdao (Feng and Liu, 2023) and Harbin (Lü and Duan, 2024) demonstrate how the transitivity framework can illuminate the ideational patterns through which city images are constructed. However, such work typically relies on small or manually compiled corpora and focuses mainly on descriptive classifications of process types, with limited exploration of participant role configurations and agency patterns.

Existing studies on regional image have largely relied on corpus-based techniques such as keyword, frequency and collocation analysis to investigate media representations of regions. While these approaches provide systematic quantitative evidence and have generated valuable insights, corpus-based discourse analysis on its own may not fully capture deeper lexical associations and latent semantic relations within large textual datasets, as noted by Mautner (2009). In this regard, text mining, as a computational technique for extracting meaningful patterns from unstructured text, offers a useful complementary perspective. It enables the identification of semantic clusters and deeper collocational relationships that go beyond surface-level quantitative regularities. Text mining has been increasingly applied in domestic studies of tourism image, including analyses of Yellow River cultural attractions (Jin, 2025) and the Mountain Yuntai scenic area (Zhou et al., 2023), yet it remains largely underutilized in research on regional image in international media discourse.

Building on these methodological considerations, the present study integrates text mining with a transitivity-based linguistic analysis to provide a more comprehensive account of Henan’s representation in Western mainstream media. Text mining is employed to trace diachronic shifts in semantic themes, while the transitivity system enables a systematic examination of discursive patterns such as “Henan does what to X” and “X does what to Henan,” thereby revealing the social roles and agency configurations assigned to the province. By applying this dual framework to a 20-year corpus spanning 2006 to 2025, the study links macro-level thematic trajectories with micro-level grammatical realizations, offering a deeper and more nuanced understanding of how Henan’s international image has taken shape within western media discourse.

## 3 Methodology

### 3.1 Theoretical framework

This study draws on Systemic Functional Linguistics (SFL) and van Leeuwen’s framework of social actor representation to examine how Western mainstream media discursively construct Henan’s regional image and how such constructions shift over time.

As one of the most influential linguistic approaches to discourse analysis, SFL, particularly the transitivity system, provides a powerful analytical framework for examining how agency, power relations, and evaluative orientations are encoded through participant roles (Wodak and Meyer, 2016). Grounded in the experiential meta-function, transitivity system models the linguistic representation of actions, events, and participants through six process types: material, mental, relational, behavioral, verbal and existential, each of which offers a distinct perspective on how discourse constructs and organizes human experience (Halliday and Matthiessen, 2004). Among these process types, material processes play a particularly prominent role in discourse by encoding concrete “doing” events in which one entity acts upon another, with the initiator of the action functioning as the Actor and the affected entity functioning as the Goal. The Actor-Goal distinction is analytically significant in news discourse, as the ways in which these roles are linguistically realized influence how agency, responsibility, and social positioning are constructed. In media reporting on Henan, the region may be represented as an Actor that initiates substantive actions affecting other entities, or as a Goal that receives impacts, undergoes changes, or becomes the object of external forces. Such linguistic realizations reveal how news discourse assigns agency and responsibility to Henan and how it positions the region within the events being reported.

To extend the analytical capacity of transitivity, the study incorporates van Leeuwen (2008) framework of Social Actor Representation, which explains how discourse activates or passivates social participants. Within this model, social actors may be foregrounded as initiators of action or backgrounded as recipients of force or assistance. This aligns directly with the Acto-Goal configurations identified through material transitivity, enabling systematic examination of whether Henan is represented as an agentive Actor or an affected Goal. Since regional image is discursively shaped through the recurrent allocation of such roles in news narratives, changes in Henan’s role distribution across Actor and Goal positions serve as an effective indicator of shifts in its construed social agency. Accordingly, the integration of the transitivity system with Social Actor Representation provides a coherent analytical foundation for assessing how Henan’s agency is distributed, negotiated, and transformed across the two corpus periods.

### 3.2 Data collection

This study employed the LexisNexis database, one of the most comprehensive repositories of international mainstream media, as the primary source for data retrieval. This research examines English-language media coverage from the United States, the United Kingdom, Australia, and Canada, whose outlets hold a dominant position in structuring Western discursive environments and, by extension, the global public sphere (Zhang and He, 2016). To ensure breadth, reliability, and representativeness, six leading newspapers were selected: *The New York Times* and *The Los Angeles Times* (the United States), *The Times* and *The Guardian* (the United Kingdom), *The Toronto Star* (Canada), and *The Australian* (Australia). These outlets were chosen not only for their wide circulation and editorial authority, but also for their agenda-setting influence and established role in shaping both national and international public discourse. Taken together, they constitute a geographically diverse and media significant sample, offering a broadly representative cross-section of Western perspectives on Henan.

Using “Henan” as the search term, this paper retrieved news articles published between 2006 and 2025 that referenced Henan in the content based on the following filtering criteria: (1) The text must include the term “Henan”; (2) The genre must be “Article” (excluding Comments, Blogs, Photos, etc.); (3) Data was downloaded yearly (2006–2025) to ensure temporal stratification. A total of 1,691 valid English news articles were collected (Table 1 for source distribution). Manual inspection showed that only two articles mentioned “Henan” in their headlines, while the majority referenced Henan-related content within broader China coverage. To ensure analytical precision, Python was used to extract all paragraphs containing “Henan,” thereby constructing the final corpus used for subsequent analysis.

**Table 1:** Sources of Henan-related news reports in Western mainstream media (2006–2025).

Corpus period	*The New York Times*	*The Los Angeles Times*	*The Times*	*The Guardian*	*The Australian*	*The Toronto Star*	Total number
2006–2015 corpus	277	110	122	178	152	65	904
2016–2025 corpus	367	40	96	210	55	19	787

For comparative purposes, the corpus was divided into two sub-corpora: 2006–2015 and 2016–2025. As explained in the Introduction, the year 2016 marks a pivotal transition in Henan’s contemporary development trajectory. Accordingly, the two periods reflect distinct phases in the province’s international positioning. The 2006–2015 corpus contains 9,195 types and 60,472 tokens, while the 2016–2025 corpus contains 7,655 types and 47,892 tokens. The corresponding type-token ratios (TTR) are 15.2 and 16.0% respectively, indicating comparable lexical diversity between the two periods.

### 3.3 Data analysis

**Figure 1:**
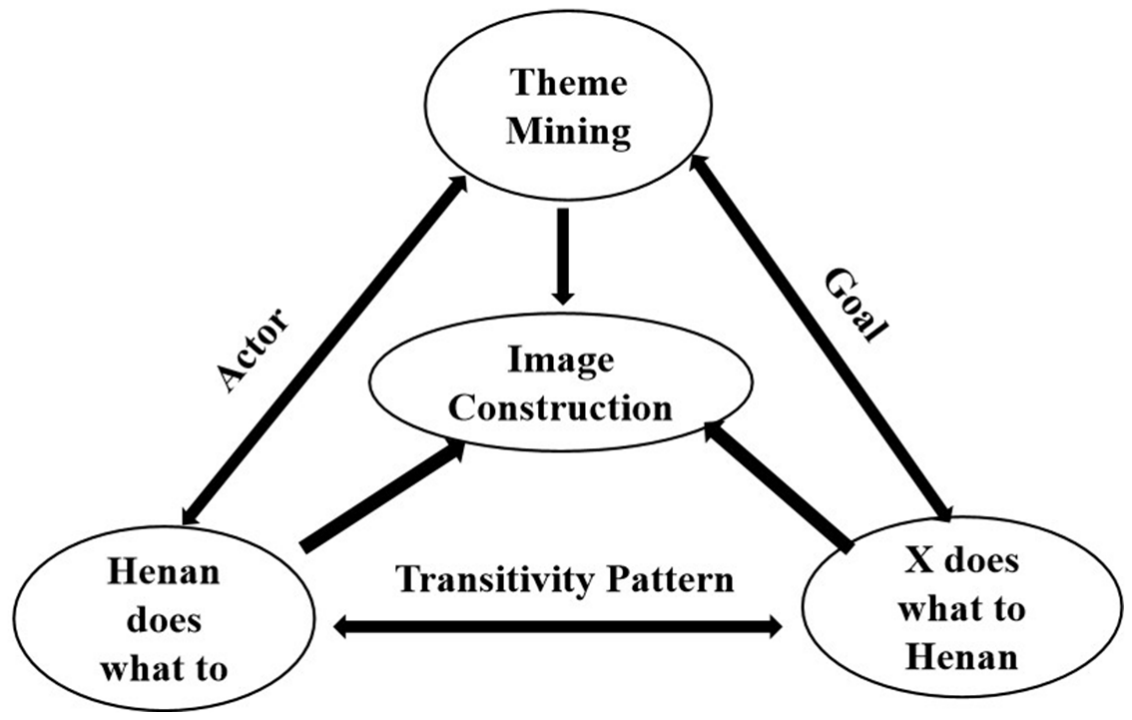
Integrated framework for diachronic analysis of Henan’s media representation.

This study adopts an integrated analytical framework combining text mining and transitivity analysis to examine the diachronic construction of Henan’s image in Western mainstream media. Text mining is used to identify and compare semantic themes across the two periods, while transitivity analysis focuses on material process patterns to reveal how Henan’s social roles are discursively configured. This dual approach enables the study to link thematic transformations with grammatical realizations. The overall analytical framework is shown in Figure 1.

To investigate thematic patterns, the study employs Leximancer 5.0, a software that uses Bayesian statistical modelling and machine-learning algorithms to detect lexico-semantic co-occurrence patterns and generate semantic network visualizations (Smith and Humphreys, 2006). Prior to analysis, the two corpora were pre-processed following a standardized four-step procedure: (1) data import: corpus texts were uploaded into the software; (2) text processing: stop words were edited in the text processing options; (3) concept optimization: eliminated semantically void concepts (e.g., “day,” “year,” “time” etc.); (4) concept normalization: merging synonymous and morphologically related terms (e.g., “kung” with “fu,” “official” with “officials” and different inflectional forms of the same verb). After preprocessing, Leximancer was employed to identify the dominant semantic concepts and themes in Western reporting on Henan and to trace their diachronic reconfiguration across the two periods.

To examine how Henan’s social roles were discursively constructed across the two decades, material process clauses were extracted using WordSmith Tools 9.0 for subsequent transitivity analysis. As shown in Table 2, two recurrent patterns were identified. In the “Henan does what to X” pattern, the verbal group realizing the Process typically follows the node word Henan, indicating that the province functions as the Actor initiating concrete actions. Conversely, in the “X does what to Henan” pattern, the verbal group generally precedes Henan, thereby positioning the province as the Goal that receives the impacts of others’ actions. To systematically retrieve these material-process instances, WordSmith Tools 9.0 was employed with a span of L5-R5 and a minimum frequency threshold of five occurrences to extract verbs collocating with “Henan” (MI ≥ 3; *t*-score≥2), which formed the basis for subsequent role classification.

**Table 2:** Instances of transitivity material process.

Material process	Actor	Process	Goal	Circumstance
Henan does what to X	Flooding in ***Henan*** Province	killed	more than 300 people	
X does what to Henan	Record-breaking rainstorms	hit	***Henan*** province in central China	in late July

Bold text indicates the node word ‘Henan’.

Based on the semantic profiles of the identified material process verbs, Henan’s Actor roles can be further categorized into Destructor, Doer, and Information Disseminator, while its Goal roles were classified into the Visited, the Destructed, and the Helped. These categories were manually annotated through iterative semantic grouping and cross-checking to ensure conceptual consistency across both corpora. After annotation, Chi-square tests were performed to assess whether the distribution of Henan’s Actor and Goal roles differed significantly between the two periods. Pearson’s chi-square was used for all roles except ‘the Helped’, for which Fisher’s exact test was applied due to low expected cell counts, respectively.

This integrated procedure allowed the study to relate theme evolution revealed through text mining with shifts in grammatical role configuration captured through transitivity analysis, thus enabling a comprehensive account of Henan’s diachronic representation in Western mainstream media.

## 4 Results

### 4.1 Diachronic shifts in thematic representation

**Figure 2:**
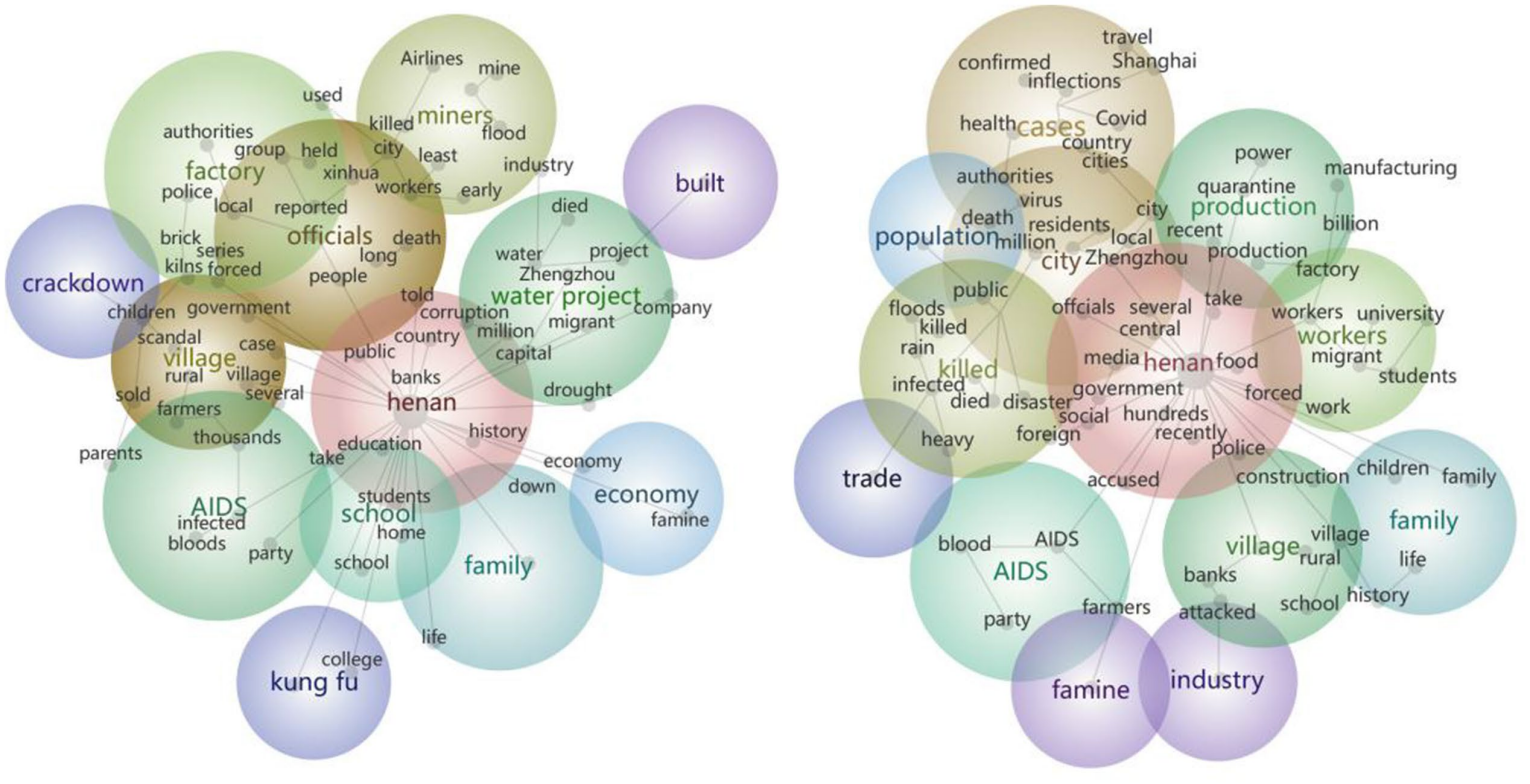
Visualized thematic networks of Henan-related coverage in Western mainstream media (Left: 2006–2015 period; Right: 2016–2025 period).

Leximancer 5.0 was employed to identify the dominant semantic themes in Western mainstream media reports on Henan across the two periods. The software extracted high-frequency themes and concepts, generating visual thematic clusters as shown in Figure 2.

The thematic analysis reveals distinct patterns in Western media coverage of Henan across the two decades. During the 2006–2015 period, the most frequent themes included Henan (1,530 hits), officials (689 hits), village (297 hits), water project (264 hits), factory (219 hits) and miners (191). These high-frequency themes generated several conceptual clusters: government policies (officials, government), rural development (village, farmers, rural), the South–North Water Transfer Project (water, project, drought) and mine accidents (factory, miners, coal, killed). During the 2016–2025 period, the most frequent themes included Henan (1,265 hits), city (568 hits), cases (343 hits), killed (212 hits), workers (194 hits) and village (192 hits). The emerging conceptual clusters in this period included government policies (officials, government), urban development (city, million, residents), the COVID-19 pandemic (cases, health, infections), flood disasters (killed, rain, floods) and commercial development (factory, economy, manufacturing).

As illustrated in Figure 2, the thematic analysis of Western media coverage of Henan between 2006 and 2025 reveals consistent discursive patterns, marked by a dual emphasis on macro-political narratives and micro-livelihood concerns. At the macro level, the recurrent prominence of the theme “officials” highlights the centrality of governmental decision-making in framing Henan, underscoring the enduring significance of provincial governance as a dominant lens through which the region is represented. At the micro level, the persistent visibility of the “family” theme across both periods indicates Western media’s sustained engagement with community-level social transformations in Henan, thereby situating everyday life within broader processes of social change.

The study further reveals a striking continuity in Western media’s preference for public emergencies and crises in Henan. In the 2006–2015 period, reporting was dominated by accounts of coal mine accidents (miners, coal, killed), brick kiln labor issues (brick, kilns, forced), and HIV infections through blood donation (AIDS, blood, infected), which rank 5th, 6th, 8th among high-frequency themes. By the 2016–2025 period, attention had shifted to COVID-19 (Covid, cases, infections), extreme weather (killed, rain, floods), rural bank crisis (rural, banks, attacked), and renewed references to the historical HIV contamination scandal (blood, AIDS), ranking 3th, 4th, 6th, 9th, respectively. Despite variations in the specific crises reported across the two periods, Western media consistently employed an “emergency-prone” framing, demonstrating discursive continuity that persistently positioned Henan within narratives of crisis.

From 2006 to 2025, Western media coverage of Henan underwent three notable thematic transformations: rural-to-urban transition, increasing economic emphasis, and decreasing cultural representation. First, the reversal from rural to urban narratives is particularly salient. Between 2006 and 2015, the “village” theme ranked third with 297 hits, reinforcing Henan’s image as an agrarian province. By contrast, during 2016–2025, its frequency declined to 192 hits (6th rank), while the “city” theme rose sharply to second place with 568 hits. This discursive shift parallels Henan’s substantive urbanization process, partially destabilizing its conventional agricultural identity in Western media portrayals.

Second, economic coverage expanded significantly. Whereas the 2006–2015 period contained only one economy-related theme (ranked 11th), the 2016–2025 period witnessed the emergence of three distinct themes: production (7th), industry (11th), and trade (12th). This thematic diversification corresponds with Henan’s structural transformation from traditional industries (brick kilns, coal mining) toward manufacturing and electronics (Foxconn, production), with occupational identity shifting correspondingly from “miners” to “workers.”

Third, cultural representation experienced relative contraction. During 2006–2015, “kung fu” (ranked 10th) served as Henan’s sole cultural symbol; however, its frequency dropped to only five hits in 2016–2025, falling below the semantic network threshold. This erosion of cultural visibility stands in stark contrast to the province’s expanding repertoire of intangible cultural heritage, revealing a form of “cultural aphasia” in international communication that undermines Henan’s potential soft power projection.

### 4.2 Diachronic shifts in Henan’s social role construction

Within the Systemic Functional Linguistics framework, material processes encode concrete actions and assign agency through the configuration of Actors and Goals. Examining how Henan occupies these participant roles therefore provides insight into the experiential meanings through which Western media discursively construct the province’s social presence over time. Table 3 presents the Chi-square test results used to assess whether the distribution of these roles changed significantly across the two periods.

**Table 3:** Diachronic variation in Henan’s social role distributions.

Role category	Social roles	2006–2015	2016–2025	Chi-square test *p*-value
Actor	Total	35 (47%)	49 (70%)	0.006**
	Destructor	20 (27%)	17 (24%)	0.707
	Doer	10 (13%)	22 (32%)	0.010*
	Information disseminator	5 (7%)	10 (14%)	0.139
Goal	Total	39 (53%)	21 (30%)	0.006**
	The visited	19 (26%)	12 (17%)	0.213
	The destructed	14 (19%)	9 (13%)	0.321
	The helped	6 (8%)	0 (0%)	0.028*

*p < 0.05, **p < 0.01.

The results reveal a statistically significant diachronic shift of Henan from being predominantly constructed as a Goal during 2006–2015 to being more frequently represented as an Actor in 2016–2025 (χ^2^ = 7.628, *p* = 0.006). During 2006–2015, Henan was slightly more frequently construed as a Goal (53%) than as an Actor (47%), suggesting that Western media tended to depict the province as the entity acted upon rather than the initiator of actions. In 2016–2025, however, this pattern reversed, with Actor roles rising markedly to 70%, substantially exceeding Goal roles (30%). As van Leeuwen (1995) argues, Actor derives social power from their ability to influence other participants within a process. Henan’s shift from a Goal-oriented representation to an Actor-dominated one therefore marks a substantive discursive transformation, signaling a strengthened portrayal of its capacity to initiate actions in Western reporting.

#### 4.2.1 Actor configurations

In order to examine how Henan’s social roles and agency were realized through different types of actions, all material clauses in which Henan functioned as Actor were grouped into three sub-categories based on their process meanings. The Destructor role was realized through verbs encoding harmful or disruptive actions such as kill or crash. The Doer role corresponded to institutionally oriented actions, typically realized through verbs such as rescue, arrest, impose, detain, or extend. The Information Disseminator role referred to clauses where Henan was construed as initiating communicative actions, typically realized through material verbs such as post and issue. These verb sets operationalized distinct experiential meanings that underpinned Henan’s enacted agency across the two periods.

In the 2006–2015 period, Henan was principally constructed as a Destructor, a role arising from frequent reports of public accidents such as coal-mine explosions, gas leaks, nightclub fires, and aviation incidents. In these cases, Henan was represented as the Destructor in material-process clauses, reinforcing its association with recurrent emergencies. By contrast, occurrences of Henan as a Doer were relatively rare, typically confined to reports of law enforcement activities and humanitarian interventions. As illustrated in Example 1, “Henan police” functioned as the Doer initiating a positive material action, rescuing individuals subjected to forced labor. Nevertheless, the limited occurrence of the constructive Doer roles (13%) was insufficient to counterbalance the overwhelmingly destructive representations (27%). Consequently, Henan’s Actor profile during this period was dominated by negative experiential meanings.

Example 1
(*Los Angeles Times*, June 15, 2007)Police in ***Henan*** province have rescued 217 people, including 29 children, who had been forced to work as slaves at brick kilns, official media reported yesterday.

In the 2016–2025 period, the distribution of the Actor roles underwent a clear reconfiguration. The Doer role became the dominant Actor type, a statistically significant increase from the previous decade (χ^2^ = 6.680, *p* = 0.010). Henan was now more frequently positioned as the initiator of concrete actions within material clauses, particularly in governance and public administration. As illustrated in Example 2, “Henan authorities” functioned as the Doer, initiating a series of preventive measures, which discursively constructed Henan as an agentive entity capable of implementing large-scale public health interventions. While the Destructor role remained present in the corpus, its relative frequency declined substantially.

Example 2
(*The Guardian*, January 7, 2022)Authorities in ***Henan*** province, China, imposed more Covid restrictions after a sharp rise in infections, limiting travel and activities in some cities or launching mass testing drives in others.

In parallel, the discursive emphasis shifted away from Henan as a site of recurrent emergencies toward Henan as a governing authority undertaking strategic and proactive actions. This transition indicates a broader redistribution of agency within Henan’s Actor role configurations, whereby the province is increasingly construed as an initiator of organized social action rather than a passive recipient of adverse events.

#### 4.2.2 Goal configurations

Henan’s Goal roles were instantiated through three recurrent sub-types, each associated with distinct sets of material process verbs. As the Visited, Henan was realized as the Goal of visiting actions, typically through process verbs such as visit or travel. As the Destructed, the province was positioned as the entity affected by adverse events, commonly through verbs such as hit or devastate. As the Helped, Henan appeared as the recipient of support in clauses involving verbs such as help or give. These configurations collectively delineated the principal ways in which the province was represented as the recipient of social action in Western mainstream media.

Across both periods, the Visited consistently emerged as the most salient Goal role. In such clauses, national leaders, journalists or tourists acted as the Actors undertaking visiting activities, while Henan, its cities, or cultural heritage sites such as the Shaolin Temple, the Longmen Grottoes and the Taoist sites were realized as the Goal. While this pattern consistently positions Henan as a location that attracts external attention across the two periods, close reading of the concordance lines reveals a diachronic shift in evaluative meaning, most notably in representations of the Shaolin Temple.

As the birthplace of Chan Buddhism and Shaolin martial arts, the Shaolin Temple functions as one of Henan’s most internationally recognizable cultural symbols and frequently serves as a key reference point for the province’s cultural heritage in Western media. In the first period from 2006 to 2015, this cultural landmark was typically represented in negative evaluative terms. As shown in Example 3, in which the Shaolin Temple was realized as the Goal of a visiting process, *The Times* depicted it as overly commercialized and theatrical. Such evaluations generate an unfavorable semantic prosody around the site, which subtly extend to Henan as a whole.

Example 3
(*The Times*, December 17, 2009)More than 1.6 million tourists visited the site in ***Henan*** province last year, each paying about £14 to watch a half-hour knockabout kung fu show and visit stores crammed with tacky martial arts-themed souvenirs.

In the second period from 2016 to 2025, Western reporting began to foreground more dialogic and collaborative forms of cultural interaction centered on the Shaolin Temple. In Example 4, the temple again occupied the Goal role in a material process of visiting, yet the experiential meaning attached to this role differed markedly from the earlier decade. Instead of being framed as commercialized or theatrical, the temple was represented as a site of artistic collaboration and spiritual engagement. Through this shift in representation, Henan is construed in more favorable evaluative terms, indicating an emerging discursive tendency to portray the province as associated with cultural depth and meaningful exchange.

Example 4
(*The New York Times*, October 11, 2018)Over a decade ago, the acclaimed Belgian choreographer Sidi Larbi Cherkaoui traveled to the Shaolin Temple in ***Henan*** Province in China to work with, and learn from, the monks there who practice Zen Buddhism and kung fu.

Among the three Goal roles, the Helped was the only one that exhibited a statistically significant diachronic change (χ^2^ = 5.922, *p* = 0.028). During 2006–2015, this role was typically instantiated in material clauses of helping or giving, with governmental ministries or other higher-level institutions functioning as the Actor and Henan as the Goal receiving support. Such constructions linguistically foreground Henan’s dependence on external assistance and align with van Leeuwen’s (1995) distinction between activation and passivation, whereby a social actor is discursively represented as lacking autonomous capacity to initiate action. This pattern was exemplified in Example 5, where the Ministry of Education functioned as the Actor in processes of giving and helping, while Henan occupied the passivated Goal position. The accompanying request for continued support further reinforced the portrayal of diminished agency during the first period.

Example 5
(The News *York Times*, March 10, 2015)“In recent years, the Ministry of Education has given ***Henan*** a lot of support and care and, on behalf of 100 million ***Henan*** people, I thank you,” said the delegate, Li Guangyu, a businessman who runs an education investment company, according to the main evening newspaper of Zhengzhou, the provincial capital. “I also sincerely ask that you continue supporting and helping ***Henan*** , and let more kids from ***Henan*** win a fair chance for an education.”

In the subsequent decade, the Helped role showed a statistically significant decline, and no instances of this role were attested in the 2016–2025 corpus. While this change suggested a diminishing tendency to depict Henan as reliant on external intervention, caution was needed in interpretation. The corpus comprised six major Western media outlets, and the absence of the Helped role did not imply that such representations had disappeared across the broader media landscape. Nonetheless, when considered alongside the marked rise of Actor roles in the same period, the decline of the Helped role indicates a broader discursive movement in which Henan is increasingly represented as an initiator of action rather than a recipient of assistance.

## 5 Discussion

This study combined text mining with a transitivity-based social actor analysis to provide a comprehensive account of how Henan has been discursively constructed in Western mainstream media over the past two decades.

The text-mining results demonstrate a marked continuity in Western media framings of Henan across the two decades. Although governance initiatives at the macro level and social transformations at the micro level appear recurrently in both periods, the most persistent and salient pattern is the sustained prominence of crisis-oriented reporting. Western media repeatedly construct the province as an “emergency-prone” region, a framing that remains remarkably stable despite broader shifts in its development trajectory. This discursive persistence aligns with Wei and Wu’s (2024) observation that French media similarly prioritize public emergencies when reporting on Henan, and it corroborates Deng’s (2017) finding that accidents and crises constitute a dominant thematic cluster in Western representations of the province. From a news-values perspective, the media’s focus on crises reflects the inherent newsworthiness of events characterized by immediacy, negativity, and human-interest appeal (Harcup and O'Neill, 2017). The endurance of this framing, however, suggests more than editorial preference, it also reflects recurring ideological orientations in Western media that foreground risk, vulnerability, and instability when depicting Chinese localities (Chen, 2023).

Beyond this continuity, the semantic themes of media coverage exhibit several notable diachronic shifts. Reporting that had previously foregrounded rural settings and agrarian concerns increasingly shifted toward urban development, infrastructure, and modernization; meanwhile, economic discourse expanded substantially as Henan’s industrial restructuring gained prominence, whereas cultural coverage experienced a relative decline. These thematic adjustments broadly mirror Henan’s developmental trajectory, particularly the province’s accelerated urbanization and economic transformation over the past decade. However, the diminishing visibility of cultural themes contrasts sharply with Henan’s exceptionally rich cultural heritage, which includes five UNESCO World Heritage Sites such as the Yinxu ruins and the Longmen Grottoes, as well as internationally recognized intangible traditions such as Shaolin Kung Fu and Tai Chi. As Chen and Sun (2023) observe, Henan’s cultural image suffers from a persistent “cultural discount” in external communication, a phenomenon that undermines the province’s soft power and international visibility, underscoring the need for more strategic cultural communication to more effectively project Henan’s cultural assets in the global arena.

The analysis of Henan’s social roles in material transitivity patterns reveals a clear diachronic shift from being primarily represented as a Goal in 2006–2015 to more frequently assuming the role of an Actor in 2016–2025. This transformation is reflected both in the transition of its dominant Actor subtype from Destructor to Doer and in the marked decline of the Helped role, jointly indicating a broader discursive reorientation away from dependency toward greater autonomy and initiative. According to van Leeuwen’s (1995) social actor theory, the Actor role indexes social power, as Actors possess the capacity to affect other participants within a social action. Henan’s transition from being primarily Goal-positioned to predominantly Actor-positioned thus represents more than a lexical redistribution, but a substantive reconfiguration of agency, foregrounding the province’s increasing capacity to initiate actions in Western reporting. This finding resonates with Lin and Miao’s (2023) analysis of American media, which similarly identifies an expansion of China’s agency over the past four decades. Taken together, these shifts indicate that Henan’s evolving representation is situated within a broader discursive trajectory in which both China and its localities are increasingly constructed as proactive social actors, reflecting their rising visibility and influence in international discourse.

Within this overall reconfiguration of agency, the Helped role undergoes a particularly noteworthy shift across the two periods. Tang’s (2021) analysis of U.S. mainstream newspapers between 2008 and 2010 found that China was frequently positioned as the Helped in transitivity patterns, ranking third among seven Goal roles in “who does what to China.” The alignment between Tang’s findings and the first-stage results of this study indicates that Henan’s construction as the Helped during 2006–2015 was not an isolated case but rather reflected a wider tendency in Western media to portray China and its sub-national entities as dependent recipients of support or intervention. Against this backdrop, Henan’s marked decline in the Helped role in 2016–2025 represents a meaningful discursive shift. Instead of being routinely cast as reliant on higher-level institutional assistance, Henan is increasingly represented as an entity capable of initiating action, reinforcing the broader upward trajectory of its discursive agency within Western media discourse.

## 6 Conclusion

Drawing on a diachronic corpus of Henan-related reports in Western mainstream media from 2006 to 2025, this study integrates text mining with the material transitivity framework to examine the evolution of semantic themes and social roles of Henan over two decades. The analysis identified both persistent frames and notable transformations, showing that while Henan continued to be recurrently associated with emergency theme, its social representation evolved from being primarily a Goal to an Actor, signaling an expansion of its perceived agency in international discourse.

Building on these findings, the study contributes on multiple levels. Empirically, the study provides a diachronic account of how Western media have represented Henan in discourse, revealing the dynamics of regional image construction over time. Theoretically, the study demonstrates that integrating text-mining with material transitivity analysis provides a systematic approach for examining how semantic patterns interact with the grammatical encoding of social roles in large-scale media corpora. Practically, the findings highlight the need for regions to enhance the global visibility of their cultural heritage, cultivate sustained narratives of modernization and governance capacity, and strengthen their international communication efforts in order to foster more balanced and multidimensional representations in global media.

While these contributions underscore the analytical and practical value of the study, several limitations merit acknowledgment. First, the dataset is restricted to six major English-language newspapers accessible through LexisNexis. While these outlets are influential agenda setters, they do not encompass the full diversity of Western media environments, thereby limiting the generalizability of the findings. Second, the transitivity analysis focuses exclusively on material processes. Although this focus is theoretically motivated by the study’s interest in social action and agency, it does not account for other process types such as relational, mental, or verbal processes, that may also shape Henan’s mediated representation. Future research could broaden the corpus to additional media genres and incorporate a wider range of transitivity processes to build a more comprehensive picture of regional image construction.

## Data Availability

The raw data supporting the conclusions of this article will be made available by the authors, without undue reservation.
